# Reducing distress in cancer patients—A preliminary evaluation of short‐term coaching by expert volunteers

**DOI:** 10.1002/pon.5111

**Published:** 2019-06-18

**Authors:** Lenneke Post, Anke I. Liefbroer

**Affiliations:** ^1^ Department of Spiritual Care Amsterdam UMC, Vrije Universiteit Amsterdam Amsterdam The Netherlands; ^2^ Faculty of Religion and Theology Vrije Universiteit Amsterdam Amsterdam The Netherlands

**Keywords:** adjustment to cancer, cancer‐related distress, emotion coaching, stepped care, well‐being

**Key points**

- This is the first evaluation of innovative short‐term coaching offered by expert volunteers, tailored to reduce cancer‐related distress.
- Coaches support people to reflect on their situation, understand their emotional state, encourage self‐management, and seek professional support if needed.
- Compared with the Dutch sample, participants beforehand reported more negative, and less positive adaptation to cancer (measured with the Mental Adjustment to Cancer scale [MAC]).
- Participants hoped mostly to improve their emotional well‐being; valued relational and reflexive skills of the coach more than substantive expertise; and experienced more positive and less negative emotions, and less negative adjustment to cancer afterwards.
- Short‐term emotion coaching by expert volunteers may be a beneficial addition to the existing stepped care approach.

## 1 BACKGROUND

Recently, the conceptualization of cancer‐related distress, and consequently of psychosocial interventions adequately dealing with this distress, is shifting. The prevailing idea that distress results from an intense emotional experience of a psychological, social, and/or spiritual nature ignores the fundamental adaptive value of emotions.^1^ Instead, cancer‐related emotions may be adaptive—potentially facilitating adjustment to cancer—if they (despite their intensity) alert, motivate, and prepare people to deal with the event of cancer and maladaptive if they perpetuate and linger across time, are extreme or instable, or interfere with one's ability to cope.^1^, ^2^ While people experiencing maladaptive emotions might be in need of specialist mental health care such as psychotherapy or pharmacotherapy, people experiencing (intense) adaptive emotions may instead benefit from nonspecialized emotion support by relatives, friends, or primary caregivers, encouraging awareness, reflection, and self‐management.^1^

This pilot study evaluates innovative short‐term coaching sessions, conducted by expert volunteers with a background in the field of psychosocial oncology, that form an intermediary between specialist mental health care and one's private support system of relatives and friends.

## 2 METHOD

### 2.1 Design

A mixed‐method pilot study was conducted aimed at studying the characteristics, expectations, and experiences of people living with cancer participating in coaching sessions by expert volunteers. Since this is a first evaluation of short‐term coaching by expert volunteers, no applicable comprehensive set of measures was available. To develop appropriate outcome measurements, participants' needs, expectations, and experiences were first qualitatively explored by means of a focus group approach with stakeholders (n = 16). Based on the results of these focus groups, a questionnaire was developed, comprising both standardized and tailor‐made scales, aimed at measuring adjustment to cancer, emotional state, and emotional, psychological, social, and spiritual well‐being.* Eligible participants (>18 y; cancer patients [all cancer types, stages, and treatment modalities], and partners of cancer patients; Dutch proficiency) completed the questionnaire shortly before (baseline) and after two or three coaching sessions (follow‐up).

### 2.2 Intervention

The intervention entails short‐term coaching sessions by expert volunteers at an independent Dutch regional center for psychosocial support, dedicated to facilitate easily accessible support for anyone living with cancer. The coaching sessions are tailored to ameliorate cancer‐related distress by supporting participants to reflect on their situation, understand their emotional state, encourage self‐management, and refer participants to professional support if needed. Coaches are volunteers with a professional background in the field of psychosocial oncology and are trained and supervised for the purpose of this intervention by the center.

### 2.3 Measures

Questionnaires comprised demographic characteristics: standardized (Mental Adjustment to Cancer scale [MAC])^3^ and tailor‐made (emotional state and emotional, psychological, social, and spiritual well‐being) patient‐reported outcome measurements.

### 2.4 Analysis

Changes over time in adjustment to cancer, emotional state, emotional, psychological, social, and spiritual well‐being were assessed via paired sample *t* tests.

## 3 RESULTS

### 3.1 Participants

From September 2016 to February 2018, 70 visitors of a Dutch center for psychosocial support took part in coaching sessions; 35 of those visitors were included and completed the baseline questionnaire; 18 participants also completed the follow‐up questionnaire. Participants not completing the follow‐up questionnaire were too distraught after the session (n = 1) or only had one coaching session (n = 16). Participants were on average 60 years old (SD = 9.5), women were overrepresented (80% at baseline; 61% at follow‐up), three quarters (74%) had or had had cancer themselves, and one quarter (26%) were partners or relatives. Compared with other cancer patients in the Netherlands and England, respondents reported more negative and less positive adaptation to cancer (MAC).^3^, ^4^ Compared with the Dutch sample, they specifically suffered to a greater extent from helplessness/hopelessness, fatalism, and anxious preoccupation and experienced to a lesser extent fighting spirit and avoidance.^3^

### 3.2 Expectations beforehand

Participants had various reasons to partake in a coaching session with an expert volunteer (see Table 1). They had questions concerning their emotional, psychological, social, or spiritual well‐being. Participants were on average mostly hoping for an improvement in their emotional well‐being, eg, in dealing with their feelings and in the processing of their emotions. Also, they hoped that their psychological well‐being would improve, by means of a better understanding of their situation, or by regaining trust in themselves; or they hoped their social well‐being would improve by feeling supported and acknowledged. For some, an improvement of their spiritual well‐being—in redeeming the purpose, enjoyment, and meaningfulness of life—was an important objective.

**Table 1:** Expectations and experiences, in averages

I hope the coaching sessions will help me to …^a^ (n = 35)	Mean^b^	SD
Better cope with my feelings (E)	3.14	.60
Process my emotions (E)	3.14	.65
Move on (P)	3.09	.82
Feel supported (S)	3.06	.59
(Re)gain trust in myself (P)	2.97	.62
Find a way to deal with my grief (E)	2.97	.75
Feel listened to (S)	2.89	.76
Better understand my situation (P)	2.89	.80
Be myself (again) (P)	2.74	.71
Be comforted (S)	2.71	.75
Ask for help (sooner) (S)	2.71	.76
Gain structure (P)	2.66	.73
Be able to enjoy the small things (again) (Sp)	2.63	.94
Experience life as meaningful (again) (Sp)	2.57	.88
Talk about my situation more easily (S)	2.46	.70
Find purpose in my life (again) (Sp)	2.43	.95
Feel less alone (S)	2.34	.94
Be at peace about questions of faith and life (Sp)	2.11	.93

I find it (very unimportant (1) – very important (5)) that the coach …^c^ (n = 18)	Mean	SD
Is trustworthy (Re)	4.61	.60
Takes time for me (Re)	4.50	.51
Listens to me (Re)	4.44	.51
Is respectful (Re)	4.39	.50
Is understanding (Re)	4.39	.50
Helps me to take a helicopter view of my situation (Rf)	4.39	.61
Supports me (Re)	4.33	.49
Helps me cope with the situation (Rf)	4.28	.46
Gives me advice (S)	4.22	.43
Helps me to get an overview (Rf)	4.11	1.19
Gives me tips and suggestions on what to do (S)	4.06	.64
Counsels me regarding how to move on (S)	4.06	.73
Indicates how to tap into my own strengths (Rf)	4.00	.69
Helps me to create inner space (Rf)	4.00	.97
Counsels me regarding existential questions (S)	3.89	1.02
Puts me at the center of attention (Re)	3.83	.86
Counsels me regarding relationships (S)	3.72	1.02
Refers me to other activities within this center (S)	3.61	.70
Refers me to (professional) help outside this center (S)	3.56	.86
Follows and supports me for a while (Re)	3.06	1.06
Counsels me regarding work (S)	2.94	1.16
Counsels me regarding faith and religion (S)	2.28	.90

a E = Emotional well‐being; P = Psychological well‐being; S = Social well‐being; Sp = Spiritual well‐being.

b This does definitely not (1)/does not (2)/does (3)/definitely does (4) apply to me.

c Re = Relational skills; Rf = Reflexive skills; S = Substantive Expertise.

### 3.3 Interaction during the coaching sessions

Retrospectively evaluating the coaching sessions, on average participants mostly valued the relational skills of their coach, such as listening, being trustworthy, respectful, and understanding (see Table 1). Moreover, participants valued the reflexive skills of their coach, like helping to gain an overall or more objective perspective on their situation. On average, participants valued these relational and reflexive skills more than substantive expertise on medical, psychological, or existential topics.

### 3.4 Outcomes of the coaching sessions

**Figure 1:**
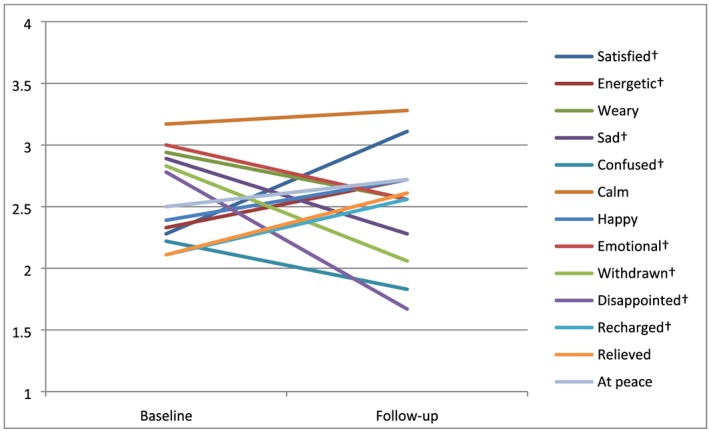
Emotions, in averages (1‐4, 1 = certainly not applicable to me; 4 = certainly applicable to me), at baseline and follow‐up (n = 18). † indicates a significant difference between baseline and follow‐up (at P < .05)

After the coaching sessions, participants improved significantly in three areas: They experienced more positive emotions, like feeling satisfied (*t*_17_ = −6.87; *P* < .01), energized (*t*_17_ = −2.12; *P* = .049), or recharged (*t*_17_ = −2.41; *P* = .028), and less negative emotions, like sadness (*t*_17_ = 3.34; *P* < .01), confusion (*t*_17_ = 2.36; *P* = .03), emotional (*t*_17_ = 2.20; *P* = .042), disappointment (t_17_ = 5.66; *P* < .01), or withdrawnness (*t*_17_ = 5.10; *P* < .01) (see Figure 1); also, respondents experienced less negative adjustment to cancer (MAC) than beforehand (Mb = 38.15 vs Mf = 31.62; *t*_12_ = 5.47; *P* < .01), especially regarding helplessness/hopelessness (Mb = 12.85 vs Mf = 10.08; *t*_12_ = 3.56; *P* < .01), fatalism (Mb = 19.31 vs Mf = 17.08; *t*_12_ = 3.71; *P* < .01), and anxious preoccupation (Mb = 23.69 vs Mf = 21.69; *t*_12_ = 3.61; *P* < .01); and finally, respondents at follow‐up felt less alone (Mb = 2.11 vs Mf = 2.78; *t*_17_ = −2.13; *P* = .048) and more listened to (Mb = 2.72 vs Mf = 3.22; *t*_17_ = −2.47; *P* = .024) than they expected or hoped for beforehand.

## 4 CONCLUSION

The results of this pilot study suggest that short‐term coaching sessions offered by expert volunteers aimed at reducing cancer‐related distress positively contribute to the adjustment to cancer, and to the well‐being of people afflicted by cancer. Our research indicates that coaching sessions—in which the coach primarily uses relational and reflexive competencies, and to a lesser extent substantive expertise on medical, social, psychological, or existential topics—not only lead to mitigation of negative emotions and improvement of positive emotions, but also to a decrease in negative, and an increase in positive adjustment to cancer.

### 4.1 Study limitations

To our knowledge, this is the first study to (quantitatively) examine the expectations, experiences and impact of short‐term coaching sessions by expert volunteers. Our research is limited by its small study sample and the lack of a control group, necessitating further research to affirm our findings.

### 4.2 Clinical implications

Despite the limitations, the results of this research suggest that people experiencing (intense) cancer‐related adaptive emotions benefit from non‐specialized support by expert volunteers, and that this support may be a beneficial addition to the existing (stepped care) approach to psycho‐oncological care. Further development of, and research into this type of intervention is therefore relevant and necessary.

## CONFLICT OF INTEREST

Authors have nothing to disclose.

## ENDNOTE

*
For reasons of brevity, this article only reports on the measurements relevant for this paper. More measurements (such as quality of life and resilience) were included in the original research.^5^
